# Hydroxychloroquine to prevent SARS-CoV-2 infection among healthcare workers: early termination of a phase 3, randomised, open-label, controlled clinical trial

**DOI:** 10.1186/s13104-023-06281-7

**Published:** 2023-02-28

**Authors:** Alejandro Llanos-Cuentas, Alvaro Schwalb, Juan Luis Quintana, Brian Delfin, Fiorela Alvarez, César Ugarte-Gil, Rosio I. Guerra Gronerth, Aldo Lucchetti, Max Grogl, Eduardo Gotuzzo

**Affiliations:** 1 Instituto de Medicina Tropical Alexander Von Humboldt, Universidad Peruana Cayetano Heredia, Av. Honorio Delgado 430, San Martín de Porres, 15102 Lima, Peru; 2 School of Medicine, Universidad Peruana Cayetano Heredia, Lima, Peru; 3 Department of Infectious Diseases, Hospital Cayetano Heredia, Lima, Peru; 4 Centro Médico Naval Cirujano Mayor Santiago Tavara, Callao, Peru; 5 School of Medicine, Universidad Científica del Sur, Lima, Peru; 6 School of Medicine, Universidad Peruana de Ciencias Aplicadas, Lima, Peru; 7 Department of Infectious Diseases, Hospital Nacional Arzobispo Loayza, Lima, Peru; 8 U.S. Naval Medical Research Unit No. 6, Lima, Peru

**Keywords:** COVID-19, Prophylaxis, Global health

## Abstract

**Objective:**

To assess the effectiveness and safety of hydroxychloroquine (HCQ) prophylaxis for the prevention of SARS-CoV-2 infection in healthcare workers (HCW) on duty during the COVID-19 pandemic.

**Results:**

A total of 68 HCWs met the eligibility criteria were randomly allocated to receive HCQ (n = 36) or not (n = 32). There were no significant differences between groups in respects to age, gender, or medical history. Eight participants met the primary efficacy endpoint of SAR-CoV-2 infection during the study period; there was no difference in incidence of SARS-CoV-2 infections between both study arms (HCQ: 5 vs Control: 3, p = 0.538). The relative risk of SARS-CoV-2 infection in the HCQ arm was 1.69 compared to the control group (95%CI 0.41–7.11, p = 0.463); due to poor participant accrual, the resulting statistical power of the primary efficacy outcome was 11.54%. No serious adverse events occurred; however, two (2/36, 5.6%) participants no longer wished to participate in the study and withdrew consent due to recurring grade 1 and 2 adverse events.

*Trial registration:* ClinicalTrials.gov ID: NCT04414241. (Registered on June 4, 2020).

## Introduction

As the novel coronavirus SARS-CoV-2 spread globally, many opted to repurpose readily available medications to combat the damaging effects of COVID-19. Among the first, the misguided use of hydroxychloroquine (HCQ) gained fast recognition early in the pandemic from preliminary data from a small study with findings suggestive of possible benefit in the treatment of COVID-19 [1, 2]. Several clinical trials (at present, over 250 trials registered on ClinicalTrials.gov) were launched to evaluate its efficacy in the treatment of COVID-19, including the well-known RECOVERY and SOLIDARITY trials [3]. Soon after, trials aimed at prevention using HCQ were abundant [4].

By the first year of the pandemic, Peru was reporting an overwhleming 1239 COVID-19 deaths per million inhabitants [5]. The healthcare workers (HCW) were among the most vulnerable groups at risk of contracting the virus. A report by the Peruvian medical governing authority showed that 3676 (from approximately 58,000) physicians had contracted COVID-19 by September 2020, of which 170 had died [6]. Similarly, by January 2021, over 6000 infections and 94 deaths were reported among nurses [7]. Additionally, 2035 midwifes had contracted COVID-19 and 21 had died [6]. As a result of the inmense death toll, and given that vaccine candidates were still under research, we sought to assess the effectiveness and safety of HCQ prophylaxis for the prevention of SARS-CoV-2 infection in HCWs on duty during the COVID-19 pandemic.

## Main text

### Materials and methods

#### Trial design

The study was a pragmatic, randomised, open-label, controled, phase 3 clinical trial evaluating two parallel arms, allocated in a 1:1 ratio, for pre-exposure prevention of SARS-CoV-2 infection in healthcare workers. The study was conducted from July to November 2020. The study consisted of eight (plus baseline) weekly visits to assess seroconversion of antibodies to SARS-CoV-2 or the presence of the virus after presenting with symptoms compatible with COVID-19. The study was approved by the Transitory National Committee of Ethics in Research (CNTEI, acronym in Spanish) as process number CNTEI-003-200. The trial is registered in the Clinical Trials Registry (NCT04414241). The study adheres to the CONSORT Guidelines.

#### Participants

We recruited participants from four public hospitals in Lima Metropolitan Area in Peru; each study site had a designated area for all study procedures. Adult healthcare workers, without evidence of SARS-CoV-2 infection (negative PCR at enrolment), working in hospital services (triage, emergency department, hospitalisation, ICU, etc.) with direct contact with patients with COVID-19 were eligible to participate. Exclusion criteria included previous (last 30 days) or current use of hydroxychloroquine, chloroquine sulfate, or azithromycin; known allergy or intolerance to hydroxychloroquine and/or chloroquine, a history of heart disease or a known history of prolonged QT syndrome; other conditions, such as Glucose-6-phosphate dehydrogenase (G6PD) deficiency, liver or kidney failure or, presence of alterations in visual acuity or field, which made participation in the study not the most benefitial for the individual.

#### Interventions

Participants in the control group were asked to adhere to standard personal protection measures against COVID-19, as recommended in the hospital. The type and quantity of personal protection equipment (PPE) were dependent on hospital and services; the trial did not provide additional PPE. No placebo was used. Patients in the intervention group were assigned HCQ and also to adhere to standard personal protection measures. The intervention consisted of a loading dose of 600 mg of HCQ orally on the first day, followed by 400 mg orally every-other-day. Participants assigned to HCQ underwent a electrocardiogram (ECG) prior to first dose to evaluate QT interval duration; if above or equal to 500 ms (Frediricia-corrected) no doses would be given. Repeat ECG were performed at week 4 and 8 of the study.

#### Randomisation

Participants were randomly allocated in a 1:1 ratio to the intervention or control group using a random number sequence generated in Excel, stratified by study site. The generated sequence was uploaded to the study project in REDCap in order to conceal sequence and correctly allocate participants after informed consent and confirming eligibility.

#### Outcomes

The primary outcome measures pertained to efficacy and safety of HCQ to prevent SARS-CoV-2 infection up to 4 weeks after randomisation. The primary efficacy endpoint was a positive polymerase chain reaction (PCR) or serological test for SARS-CoV-2 and the primary safety endpoint was grade 3 or greater adverse event during follow-up. The secondary outcome concerned the tolerability of the prophylactic treatment and was measured as the proportion of participants opting out of treatment due to the presence of grade 1 or 2 adverse events.

#### Sample size

Based on the scarce information available at the time of the design of the study, we assumed that the risk in HCWs of contracting COVID-19 was 30%. Furthermore, a 50% relative reduction in the incidence of SARS-CoV-2 infection was considered to be the minimum desirable for this intervention. For a 50% reduction, 95% confidence (type 1 error, α = 0.05), 80% power, and with a potential loss to follow-up of 20%, it was estimated that 160 participants per arm were needed.

#### Statistical analysis

The analysis was by an intention-to-treat approach, including all participants who were randomised to a study arm. Categorical variables describing baseline data were presented in frequencies and percentages, and continuous variables with means and standard deviation (SD). Chi-square or Fisher’s exact test were used to evaluate differences in categorical variables between arm groups; for continuous variables, a Student’s t-test was used. For the primary efficacy endpoint an incidense density and ratio was calculated. Frequencies were reported for the primary safety endpoint and secondary outcome. Data analysis was performed in Stata SE 16.1 (StataCorp, USA).

## Results

**Fig. 1:**
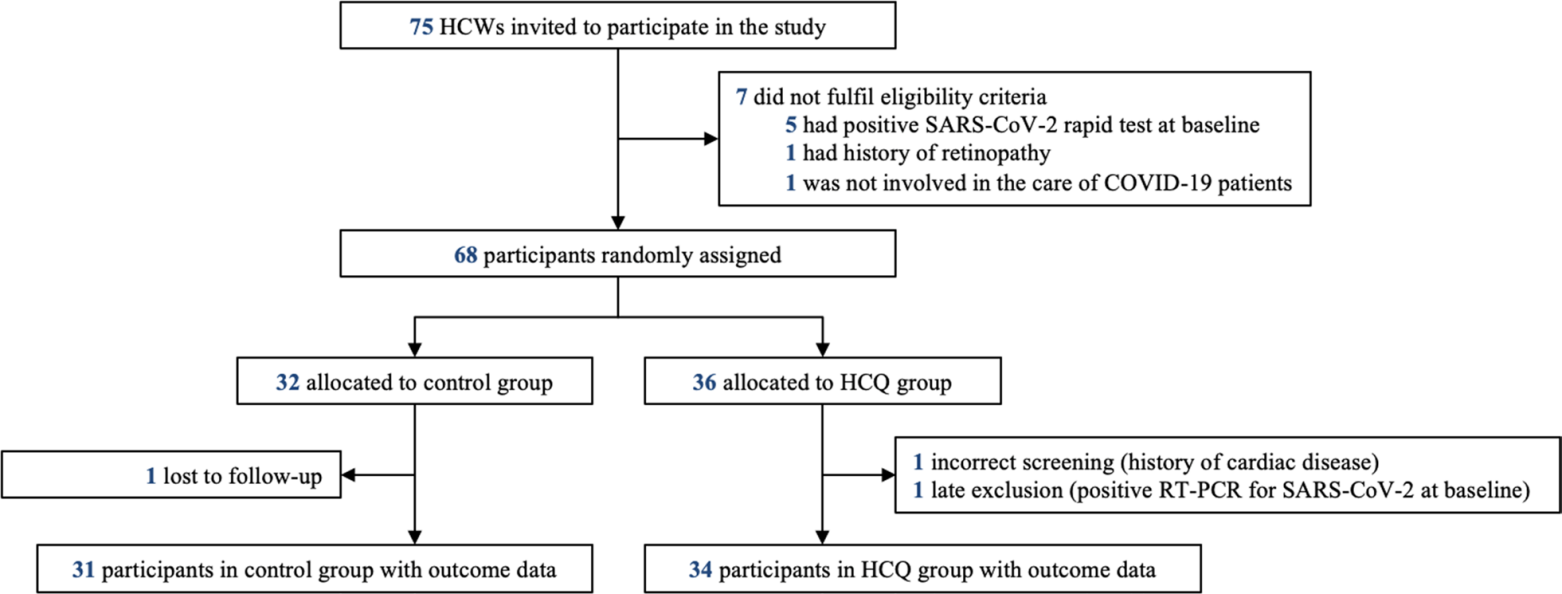
Screening and participant flow diagram. *HCW* healthcare workers, *SARS-CoV-2* severe acute respiratory syndrome coronavirus 2, *COVID-19* coronavirus disease 2019, *HCQ* hydroxychloroquine

Out of four planned study sites only three opted to enrol and follow participants. A total of 75 HCWs from were invited to participate, of which only 68 met the eligibility criteria and were randomly allocated (Fig. 1). Most participants were female (59%), with a mean age of 39.2 years (SD:9.36). There was greater participation of medical staff (57%). Comparative demographic information, medical history, and baseline exposure data between both arms are shown in Table 1. There were no significant differences between groups in respects to age, gender, or medical history. A difference was found with the use of the face shield, with the HCQ group reporting greater use compared to the control group (p = 0.025).

**Table 1:** Baseline characteristics of participants

Variable	HCQ (n = 36)	Control (n = 32)	P value^*a*^
Site			0.639
HCH	12 (43.8)	14 (33.3)	
CMN-CMST	17 (53.1)	22 (61.1)	
HNAL	1 (3.1)	2 (5.6)	
Female sex	20 (55.6)	20 (62.5)	0.561
Age, mean, years (SD)	39.14 (1.53)	39.28 (1.72)	0.951
Type of HCW			0.309
Medical staff	23 (63.9)	16 (50.0)	
Nursing staff	6 (16.7)	5 (15.6)	
Technical staff	6 (16.7)	11 (34.38)	
Nursing assistant	1 (2.8)	0	
BCG vaccination	32 (88.9)	31 (96.9)	0.208
Diabetes mellitus	1 (2.8)	0	0.342
Obesity	3 (8.3)	2 (6.3)	0.743
Arterial hypertension	3 (8.3)	2 (6.3)	0.743
Asthma	3 (8.3)	4 (12.5)	0.573
PPE use in last shift			
N95/Respirator	35 (97.2)	31 (96.9)	0.933
Surgical mask	27 (75.0)	26 (81.3)	0.535
Goggles	31 (86.1)	23 (71.9)	0.147
Face shield	31 (86.1)	20 (62.5)	0.025
Overalls	27 (75.0)	21 (65.6)	0.397
Gown	35 (97.22)	28 (87.5)	0.125
Disposable boots	31 (86.1)	26 (81.3)	0.587
Gloves	34 (94.4)	28 (87.5)	0.314
Exposure during last week, mean, days (SD)	2.89 (0.24)	3.25 (0.31)	0.359
Daily exposure per day, mean, hours (SD)	10.14 (1.12)	8.56 (0.71)	0.252
Household COVID-19 contact	4 (11.1)	3 (9.4)	0.814
Mode of transport to hospital			0.937
Private transport	14 (38.9)	13 (40.6)	
Taxicab	5 (13.9)	4 (12.5)	
Public transport	14 (38.9)	11 (34.4)	
Other	3 (8.3)	4 (12.5)	

Values are n (%), unless noted otherwise

*HCQ* hydroxychloroquine, *HCH* Hospital Cayetano Heredia, *CMN CMST* Centro Médico Naval—Cirujano Mayor Santiago Tavara, *HNAL* Hospital Nacional Arzobispo Loayza, *SD* standard deviation, *BCG* Bacillus Calmette-Guerin, *BMI* body mass index, *PPE* personal protective equipment

^a^Chi-square test for categorical variables and Student’s t-test for continuous variables

^b^Fisher’s exact test

Eight participants met the primary efficacy endpoint of SAR-CoV-2 infection during the study period: four by rapid serological test and four by PCR from nasopharyngeal swab prompted by the presence of symptoms. Six infections were reported in participants from CMN-CMST, and two from HCH. There was no difference in incidence of SARS-CoV-2 infections between both study arms (HCQ: 5 vs Control: 3, p = 0.538). The relative risk of SARS-CoV-2 infection in the HCQ arm was 1.69 compared to the control group (95% CI 0.41–7.11, p = 0.463). Due to poor participant accrual, the resulting statistical power of the primary efficacy outcome was 11.54%.

Regarding drug safety, no serious adverse events occurred. However, a grade 1 adverse cardiac event was reported in one participant who presented with a 48 ms increase in the QTc interval (Baseline: 389 ms) associated with palpitations. Palpitations occurred after receiving 15 doses of HCQ. Based on the recommendation of the study cardiologist, the regimen was modified to receive HCQ every 3 days. A week after, in the control ECG, a QTc interval of 399 ms was observed and the study regimen was continued. Regarding the tolerability of HCQ prophylaxis, two (2/36, 5.6%) participants no longer wished to participate in the study and withdrew consent due to recurring grade 1 and 2 adverse events (mainly headache and dyspepsia). The list of reported adverse event reports is shown in Table 2.

**Table 2:** Adverse events reported by HCQ group

Adverse event	Frequency (n = 34)
Headache	11 (32.3)
Dyspepsia	10 (29.4)
Nausea	8 (23.5)
Myalgia	8 (23.5)
Anorexia	5 (14.7)
Dizziness	3 (8.8)
Low back pain	2 (5.8)
Palpitations	1 (2.9)

Values are n (%)

*HCQ* hydroxychloroquine

## Discussion

We observed that there is no difference in SARS-CoV-2 infection incidence between the group that received HCQ prophylaxis and the control group. Despite the low statistical power of our study, this finding aligns with other trials evaluating the effectiveness of HCQ to prevent SARS-CoV-2 infection or treat COVID-19 [8, 9].

At the beginning of the pandemic, a call was made for well-designed, robust trials to combat the growing number of observational studies and anecdotal evidence that hinted at the benefits of HCQ. Among the first published, a trial on post-exposure (within 4 days) HCQ prophylaxis found no difference in the incidence of illness compatible with COVID-19 [10].The strength of the study was the clear exposition to SARS-CoV-2, subcategorised as moderate or high [10]; thus, allowing the researchers to observe if there was resolution of clinical symptoms or prevention of severe disease. A post-exposure non-randomised trial among HCWs also failed to show a substantial benefit associated with the use of HCQ [11]. For many researchers the intended purpose of HCQ was as pre-exposure prophylaxis; however, other trials focusing on pre-exposure and HCWs, also found no clinical benefit from the use of HCQ [12, 13]. Our findings add, albeit slightly, to the growing evidence of no benefit in the use of HCQ against SARS-CoV-2 infection. Furthermore, result reporting has been notably deficient with COVID-19 trials, especially in the case of discontinued trials [14]; thus, presenting these findings add transparency to the topic.

Fortunately, the trials did not report an increase in serious adverse events, in particular cardiac, associated with the use of HCQ [10–13]. Concerns due to prolongation of QT interval and potentially lethal arrhythmias have been mostly associated with the concomitant use of HCQ and azithromycin or other contraindications to HCQ [15], which is why HCWs who had received these drugs were excluded from participating. Lower grade adverse events due to HCQ were common, and as shown in our findings, appear to be an obstacle in participant adherence and retention in the study [10–13].

Within the first 100 days of the pandemic, a vast number of trials had already been registered on ClinicalTrials.gov and the World Health Organization Inter- national Clinical Registry Platform [14]. Our study is one of many evaluating HCQ for COVID-19 prevention, which ultimately resulted in excessive duplication of research in a pharmacological intervention that was not beneficial [16]. Although this was done to rapidly obtain a therapeutic agent against SARS-CoV-2 infection, many of the trials—including ours—were small (< 500 sample size) and ultimately contributed to research waste or misleading conclusions [16]. Adaptive and pragmatic platform trials that were embedded in clinical care, such as SOLIDARITY or RECOVERY, were key in generating evidence with ample collaboration across multiple sites, allowing for a large sample size and representativeness of patients [16]. Looking forward, having these research platforms in place, with sites ready to enrol and speedy ethical approval processes, would increase pandemic preparedness. Additionally, robust evidence from such trials, arriving at a prompt time would limit and discourage self-medication of untested drugs.

## Limitations

The main limitation of our study was the poor participant accrual. Despite the pragmatic design of the trial, we only managed to enrol 21% (68/320) of the intended sample size, from three out of four planned study sites. This design was intended to increase generalisability of the findings and, if beneficial, a rapid uptake into clinical practice [17]. One major reason why recruitment was difficult was that the study period coincided with the publishing of studies that showed no benefit associated with the use of HCQ. Of note, HCWs in our study were seeing first-hand the (lack of) effect of HCQ in hospitalised patients with COVID-19. In addition, safety concerns about QT prolongation (and even Torsades de Pointes) were prominent among HCWs [15, 18]. As a result, after the initial influx of participants, accrual became difficult as HCWs were no longer interested in participating in a trial testing a drug that no longer had any perceivable clinical equipoise.

## Data Availability

The datasets used and/or analysed during the current study available from the corresponding author on reasonable request.

## Abbreviations

BCG: Bacillus Calmette-Guerin
CMN-CMST: Centro Médico Naval—Cirujano Mayor Santiago Tavara
CNTEI (acronym in Spanish): Transitory National Committee of Ethics in Research
COVID-19: Coronavirus disease 2019
ECG: Electrocardiogram
G6PD: Glucose-6-phosphate dehydrogenase
HCH: Hospital Cayetano Heredia
HCQ: Hydroxychloroquine
HCW: Healthcare worker
HNAL: Hospital Nacional Arzobispo Loayza
ICU: Intensive care unit
PCR: Polymerase chain reaction
PPE: Personal protective equipment
SARS-CoV-2: Severe acute respiratory syndrome coronavirus 2
SD: Standard deviation
